# Pullulan Nanoparticles as Prebiotics Enhance the Antibacterial Properties of *Lactobacillus plantarum* Through the Induction of Mild Stress in Probiotics

**DOI:** 10.3389/fmicb.2019.00142

**Published:** 2019-02-06

**Authors:** Liang Hong, Whee-Soo Kim, Sang-Mok Lee, Sang-Kee Kang, Yun-Jaie Choi, Chong-Su Cho

**Affiliations:** ^1^Department of Agricultural Biotechnology, Seoul National University, Seoul, South Korea; ^2^Institutes of Green-bio Science & Technology, Seoul National University, Pyeongchang, South Korea; ^3^Research Institute of Agriculture and Life Sciences, Seoul National University, Seoul, South Korea

**Keywords:** probiotics, prebiotics, pullulan nanoparticles, internalization, plantaricin

## Abstract

Synbiotics, which are the combination of probiotics and prebiotics, have recently attracted attention because of their synergistic net health benefits. Probiotics have been used as alternatives to antibiotics. Among the probiotics, *Lactobacillus plantarum* (LP) has shown strong antimicrobial activity against *Escherichia coli K99*, a major livestock pathogen. In this study, we aimed to investigate the antimicrobial activity of phthalyl pullulan nanoparticle (PPN)-treated LP. Interestingly, when PPNs were added to LP, the PPNs were internalized into the LP through an energy-dependent and galactose transporter-dependent mechanism. Additionally, more plantaricin, a natural antibacterial peptide, was secreted from PPN-treated LP than from untreated or pullulan-treated LP. Furthermore, antimicrobial activity against Gram-negative *Escherichia coli K99* and Gram-positive *Listeria monocytogenes* by PPN-treated LP was higher than those of untreated or pullulan-treated LP. It is thought that the enhanced antimicrobial properties of the PPN-treated LP are due to intracellular stimulation. Overall, this research provides a new method of producing plantaricin in LP through intracellular stimulation by internalized PPNs.

## Introduction

According to the World Health Organization (WHO), the use of antibiotics as growth promoters for livestock is a major cause of antibiotic resistance. Antibiotic resistance affects not only livestock health but also human health (World Health Organization, 2000). Therefore, finding alternatives to antibiotics and addressing drug resistance have become important issues for scientists (de la Fuente-Nunez et al., 2012; Allen et al., 2014). Recently, several studies have demonstrated the potential of probiotics are potential candidates as antibiotic alternatives due to their ability to inhibit bacterial colonization on the gut barrier or to directly kill pathogens through their secreted bacteriocins (Gillor et al., 2008). Therefore, there have been many attempts to increase the production of bacteriocins, including biological and physical methods. A biological engineering strategy as one of biological methods enhanced production of bacteriocins in probiotics with higher stability and good characteristics (Papagianni and Anastasiadou, 2009); however, the method is very complex, and consumers are increasingly concerned about genetically modified products. The physical methods used to optimize the production of bacteriocins include changing pH, temperature, pressure, oxygen content, and incubation time during probiotic culture (Arokiyamary and Sivakumaar.,2011). Interestingly, in our previous studies (Cui et al., 2018; Kim et al., 2018), pediocin production in *Pediococcus acidilactici* (PA) was markedly enhanced through intracellular stimulation by internalized inulin nanoparticles used as a synbiotic.

Among the probiotics, *Lactobacillus plantarum* (LP) is a versatile and abundant microorganism found in several environments ranging from food to animal gastrointestinal tracts (de Vries et al., 2006). It is also known that some strains of LP are capable of producing several natural antimicrobial substances, such as bacteriocins and organic acids (lactic acid and acetic acid), thereby inhibiting competitors in the same niche (Todorov et al., 2011; Reis et al., 2012). It was previously reported that LP 177 isolated from pig intestines exhibited strong antibacterial activity against *E. coli K99*, which can cause bacterial diarrhea in pigs (Yun, 2007; Seo, 2012).

Prebiotics used as non-digestible food additives beneficially affect the host by selectively stimulating the growth and/or activity of a limited number of microorganisms in the colon (Gibson and Roberfroid, 1995). Most prebiotics are inulin-based fructose oligomers or galacto-oligosaccharides (Kneifel, 2000). Among potential prebiotic compounds, pullulan has long been applied to food additives (Cheng et al., 2011). Pullulan is an α-1,6 linked polymer of maltotriose subunits and is secreted by the fungus *Aureobasidium pullulans* (Catley et al., 1986). Due to its high molecular weight and slow hydrolysis by α-amylase and glucoamylase, pullulan is considered to be a non-digestible carbohydrate (Leathers, 2003). It was previously reported that pullulan fermented by the microbiota can alter the composition of the intestinal microbiota (Sugawa-Katayama et al., 1994).

In recent years, many researchers have begun to synthesize and apply drug delivery systems based on pullulan-based self-assembled nanoparticles (Na et al., 2003; Jeong et al., 2006; Zhang et al., 2010). By contrast, our synthetic PPN application is not a drug or gene carrier but a new type of prebiotic.

One of the simplest ways to synthesize polymeric nanoparticles is the self-assembly of hydrophobically modified hydrophilic polymers. Self-assembled polymeric nanoparticles, consisting of a hydrophobic core and a hydrophilic shell, have been used as promising drug carriers because they can be rapidly internalized by mammalian cells after loading drugs into their hydrophobic cores (Zhang et al., 2008).

In this study, we are aimed to investigate the antimicrobial activities of phthalyl pullulan nanoparticle (PPN)-treated LP. We synthesized and characterized PPNs to develop a new type of prebiotic for LP. In addition, we checked whether the internalization of PPNs by LP led to enhanced antimicrobial activity by LP against Gram negative bacteria *Escherichia coli K99* and Gram positive bacteria *Listeria.monocytogenes* (LM) than LP or pullulan alone by antimicrobial assays. We further validated the mechanism of the antimicrobial activity of PPN-treated LP by the internalization of PPNs by LP.

## Materials and Methods

### Materials

Pullulan used in this study was purchased from Shandong Freda Biotechnology Co., Ltd. (Shandong, China), and other chemicals were purchased from Sigma-Aldrich (St. Louis, MO, United States). Lysogeny broth (LB), LB agar, De Man, Rogosa and Sharpe agar (MRS) broth, MacConkey agar, and brain heart infusion (BHI) broth were purchased from BD Difco (Sparks, MD, United States) for bacterial culture.

### Synthesis of PPNs

Phthalyl pullulan nanoparticles were synthesized according to a previously described method,(Na and Bae, 2002) with a slight modification. One gram of pullulan was dissolved in 10 ml of dimethyl formamide (DMF), and 0.1 mol-% dimethylaminopyridine per pullulan sugar residue was added to the solution as a catalyst., and then phthalic anhydride was added to the above solution at different molar ratios per pullulan, including 6:1 (phthalic anhydride: pullulan) (named PPN1), 9:1 (phthalic anhydride: pullulan) (named PPN2), and 12:1 (phthalic anhydride: pullulan) (named PPN3), to produce PPNs with different degrees of substitution of phthalic groups. The reaction was performed at 54°C for 48 h under nitrogen. The produced PPNs were dialyzed first in DMF to remove unreacted phthalic anhydride and then in distilled water at 4°C for 24 h to form self-assembled nanoparticles of phthalyl pullulan. The unreacted pullulan was removed after ultra-centrifugation of prepared PPNs. Finally, the PPNs were freeze-dried and stored at -20°C until use.

### Characterization of PPNs

The content of the phthalyl groups in PPNs was confirmed by 600 MHz ^1^H-nuclear magnetic resonance (NMR) spectroscopy (AVANCE 600, Bruker, Germany). The surface topography of PPNs was analyzed using a field-emission scanning electron microscope (FE-SEM) with SUPRA 55VP-SEM (Carl Zeiss, Oberkochen, Germany). The PPNs were mounted onto stubs with adhesive copper tape and coated with platinum under a vacuum using a coating chamber (CT 1500 HF, Oxford Instruments, Oxfordshire, United Kingdom). The sizes of the nanoparticles were measured with a dynamic light scattering (DLS) spectrophotometer (DLS-7000, Otsuka Electronics, Japan). The zeta potential of the nanoparticles was measured with an electrophoretic light scattering (ELS) spectrophotometer (ELS-8000, Otsuka Electronics, Japan).

### Confirmation of Internalization of PPNs by LP

First, the fluorescence isothiocyanate (FITC)-labeled PPNs were prepared as follows. Five mg of FITC was mixed with 100 mg PPNs or pullulan dissolved in 2 ml dimethyl sulfoxide (DMSO). After stirring for 4 h in an opaque tube at room temperature, the products were dialyzed against distilled water at 4°C for 24 h. Finally, FITC-labeled PPNs and pullulan were lyophilized and stored at -20°C until use.

To observe the internalization of PPNs and pullulan by probiotics, LP 177 (2.0 × 10^6^ CFU/ml) was inoculated into 1 ml of MRS broth, treated with 0.5% (w/v) FITC-PPNs or FITC-pullulan, and incubated for 2 h at 37°C. The samples were then washed with PBS and analyzed by flow cytometry and confocal laser scanning microscopy (CLSM) (SP8X STED, Leica, Wetzlar, Germany). To confirm the internalization of nanoparticles into the probiotics, LP treated with FITC-PPN3 was observed by Z-section mode in CLSM.

To confirm the temperature-dependent internalization of nanoparticles, three separate cultures of LP were treated with 0.5% (w/v) FITC-PPN3 and incubated at 4, 20, and 37°C for 2 h. The samples were further washed with PBS and analyzed by flow cytometry and CLSM. To confirm further the transporter-dependent internalization of nanoparticles into probiotics, and glucose, galactose, fructose and PPN3 were used as blocking agents. LP (2.0 × 10^6^ CFU/ml) was inoculated into 1 ml of PBS and pre-treated with 10% (w/v) glucose, galactose, fructose or PPN3 for 10 min at 37°C before treatment with 0.5% (w/v) FITC-PPN3. After 2 h of incubation at 37°C, the samples were washed three times with PBS, and the internalization of PPN3 was analyzed by flow cytometry and CLSM.

### Bacterial Cultures

*Escherichia coli (E. coli)* K99 and *Listeria monocytogenes* (LM) were used as representative Gram-negative and Gram-positive pathogens, respectively. MRS, LB, and BHI broths were used for LP 177, *E. coli* K99, and LM, respectively. All bacteria cultures were incubated at 37°C in a shaking incubator (250 rpm) for 24 h prior to experimental procedures or stored at -70°C in 15% glycerol for further use.

### Co-culture Assay and Agar Diffusion Test for Antimicrobial Ability

Antimicrobial activity of LP against *E. coli* and LM was determined using co-culture assays (Ditu et al., 2011) and agar diffusion tests (Driscoll et al., 2012), with some modifications. To compare the antimicrobial activity of LP against *E. coli* by co-culture assay, 2.0 × 10^6^ CFU/ml of *E. coli* was co-cultured with 2.0 × 10^5^ CFU/ml LP treated with or without 0.5% (w/v) PPNs or pullulan in MRS broth for 8 h at 37°C under aerobic conditions in a shaking incubator (250 rpm). The antimicrobial activity was determined by the survival rate of *E. coli*. The co-cultured samples were spread on MacConkey agar and incubated for 24 h at 37°C, and the number of *E. coli* colonies was counted. The antimicrobial activity of LP against LM was also determined by co-culture assay. LP and LM were co-cultured in BHI broth under similar conditions as described above. Finally, the co-cultured samples were spread on Oxford agar, and the number of LM colonies was counted.

The agar diffusion test was used to determine whether the cultured medium of LP treated with or without PPNs and pullulan was able to inhibit the growth of pathogens on an agar plate. First, 100 μl *E. coli* stock (2.0 × 10^8^ CFU/ml) was spread onto LB agar. A paper disk was placed on the *E. coli*-spread plate, then 120 μl 8 h-cultured LP media of LP treated with or without (0.5% w/v) PPNs and pullulan was dropped onto the paper disk. After drying at room temperature, the plate was cultured overnight at 37°C. The zone of inhibition of *E. coli* growth was used as a direct measurement of antimicrobial activity. The same protocols were followed to test the inhibitory effect of LP treated with or without 0.5% (w/v) PPNs or pullulan on LM growth on BHI agar plates.

To confirm plantaricin activity, agar diffusion tests of LP against *E. coli* and LM were performed using the same protocols described above after the culture medium was treated with 1 mg/ml proteinase K and incubated at 37°C for 2 h, and then each culture supernatant was heated at 100°C for 30 min.

### Analysis of the Growth Conditions of LP

After treatment of LP with or without PPNs or pullulan as described above, the growth characteristics of the LP were checked by measuring the pH of growth medium and viable cell counts at the indicated time points.

### Protein Isolation and Identification by SDS–PAGE

Plantaricin was isolated and purified as described in a previous study(Song et al., 2014) with some modifications. Supernatants from the cocultured medium were stirred with ammonium sulfate (80% saturation) for 2 h at room temperature. The precipitated proteins, collected by centrifugation, was dissolved in citrate phosphate buffer (50 mM) and desalted by dialysis (1 kDa cut-off membrane, Spectrum Lab, United States). Proteins were lyophilized and stored at 4°C for further analyses.

Sodium dodecyl sulfate-polyacrylamide gel electrophoresis (SDS–PAGE) was used to observe the isolated plataricin.

### Analysis of Stress Response and Plantaricin Genes by Quantitative Real-Time PCR

RNA extraction was performed using the TRIzol^®^ Max^TM^ Bacterial RNA Isolation Kit purchased from Thermo-Fisher Scientific Inc. (Waltham, MA, United States). Total RNA extraction was conducted according to the manufacturer’s instructions. LP was treated with or without PPNs or pullulan as described above. After the isolation of RNA, cDNA was synthesized from 1 μg RNA using ReverTra Ace^®^ qPCR RT Master Mix with gDNA Remover purchased from TOYOBO CO., LTD (Dojima, Osaka, Japan). Quantitative real-time PCR (qRT-PCR) was performed with SYBR qPCR Mix using one-step real-time PCR. The primers are listed in Table 1. Relative gene expression was calculated using the -2ΔΔCt method. The target gene expression was normalized to the relative expression of 16s rRNA as an internal control in each sample.

**Table 1:** Primers used in this study.

Gene	Primer sequence (5′-3′)	Size (bp)
planS	F:GCCTTACCAGCGTAATGCCC	450
	R:CTGGTGATGCAATCGTTAGTTT	
dnaK	F:ATTAACGGACATTCCAGCGG	600
	R:TTGGCCTTTTTGTTCTGCCG	
dnaJ	F:GGAACGAATGGTGGCCCTTA	474
	R:CTAGACGCACCCACCACAAA	


## Results

### Synthesis and Characterization of PPNs

**FIGURE 1:**
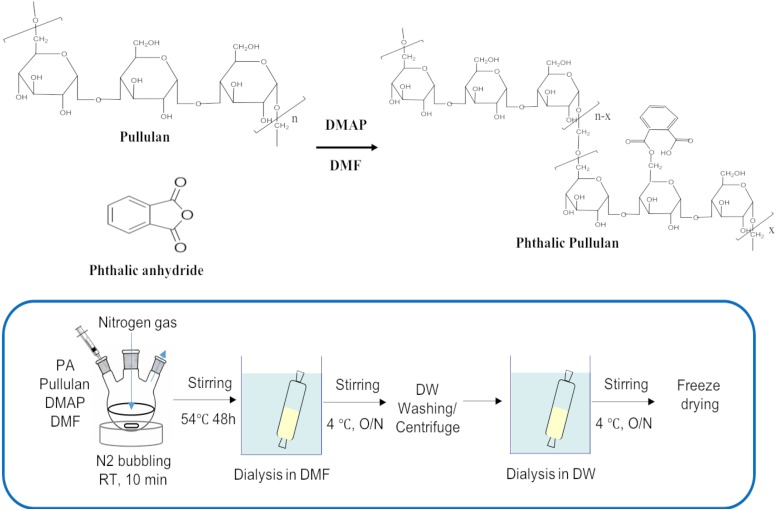
Chemical reaction scheme for the synthesis of PPNs (PPNs: phthalyl pullulan nanoparticles).

**FIGURE 2:**
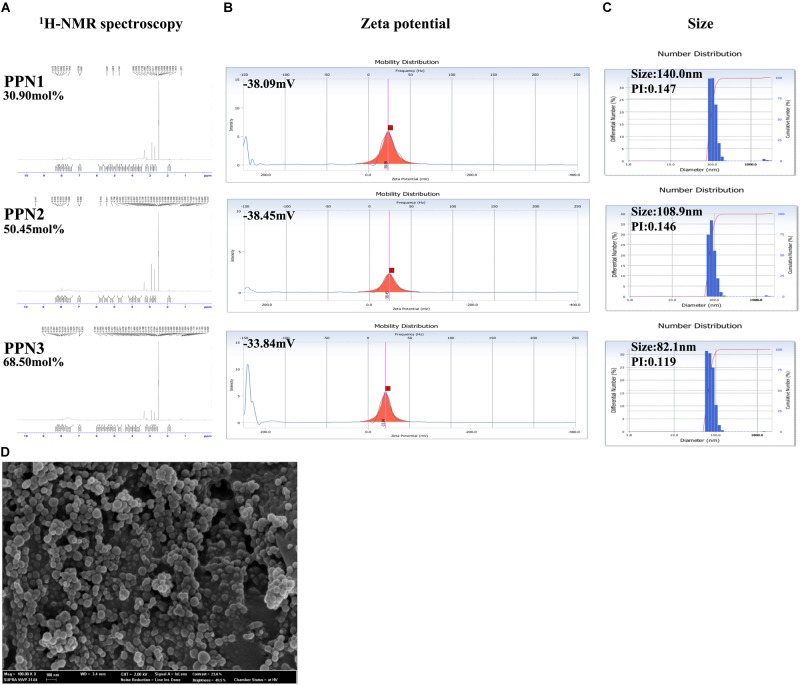
Characteristics of PPNs. Calculation of mol.-% phthalic acid in PPNs by ^1^H-NMR spectroscopy **(A)**. Measurement of the zeta potential of PPNs by ELS **(B)** and size by DLS **(C)**. Morphologies of PPNs observed by SEM **(D)**. Magnification: 100,00K, scale bar = 100 nm. (PPNs: phthalyl pullulan nanoparticles, H-NMR: nuclear magnetic resonance, ELS: electrophoretic light scattering, DLS: dynamic light scattering, SEM: scanning electron microscope).

The reaction scheme of PPN synthesis is shown in Figure 1. The degree of substitution of phthalic moieties in pullulan was confirmed by ^1^H-NMR spectroscopy (Figure 2A) and calculated by determining the ratio of phthalic acid protons (7.4–7.7 ppm) to sugar protons (C_1_ position of α-1,6 and α-1,4 glycosidic bonds, 4.68 and 5.00 ppm, respectively) as described by Tao et al. (Tao et al., 2016). According to the degrees of substitution of phthalic acid, the PPNs were named as follows: PPN1 (DS: 30.90 mol. -%), PPN2 (DS: 50.45 mol. -%) and PPN3 (DS: 68.50 mol. -%). Using SEM, PPN3 (Figure 2D) was determined to be spherical and sized between 100 and 150 nm. The sizes of nanoparticles measured by DLS were 140.0, 108.9, and 82.1 nm for PPN1, PPN2, and PPN3, respectively (Figure 2C), indicating that the particles sizes decreased with an increase in the number of conjugated phthalic acid groups in pullulan. Furthermore, the surface charges of the PPNs, measured by ELS, were -38.09, -38.45, and -33.84 mV for PPN1, PPN2, and PPN3, respectively (Figure 2B). Due to the unreacted carboxyl groups in phthalic acid, the PPNs showed negative zeta potential.

### Internalization of PPNs by LP

**FIGURE 3:**
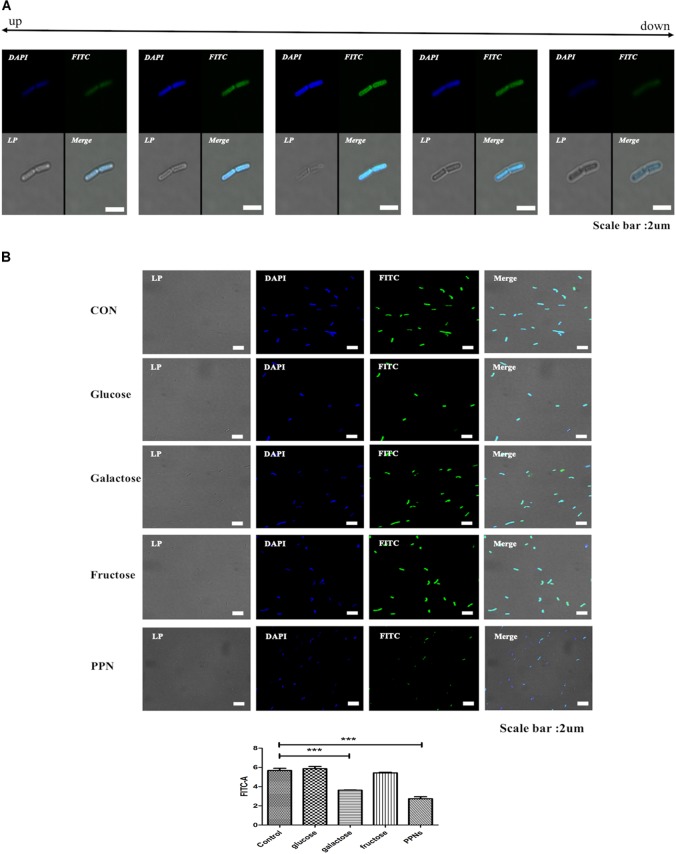
Analysis of the internalization of PPNs by LP. FITC-PPNs are shown in green, and LP was stained blue with DAPI. The internalization of PPNs after 2 h of treatment was quantified by FACS and statistically analyzed. Z-section images show the internalization of corresponding PPNs into LP **(A)**. Analysis of the internalization of PPNs by LP depending on transporters **(B)**. LP, pre-incubated with 10% (w/v) glucose, fructose, galactose or PPN3 was treated with 0.5% (w/v) FITC-PPN3 for 2 h at 37°C, and internalization was observed by CLSM and FACS. Confocal and FACS data are representative of three independent experiments, and the average values are presented as the mean ± SEM of three independent FACS experiments in a bar chart. Statistical significance was analyzed between each group by one-way ANOVA and Tukey’s *t*-test (^∗∗∗^*p* < 0.001). Scale bar = 10 μm. (LP: *Lactobacillus plantarum*, PPN: phthalyl pullulan nanoparticle, CLSM: confocal laser scanning microscopy, FACS: fluorescence-activated cell sorting, FITC: fluorescein isothiocyanate, DAPI: 4′,6-diamidino-2-phenylindole).

To confirm the internalization of PPNs by LP, PPNs were conjugated to fluorescence isothiocyanate (FITC). The internalization of FITC-PPNs by LP was analyzed by CLSM and quantified by fluorescence-activated cell sorting (FACS). It was observed by CLSM that FITC-PPNs and FITC-pullulan were able to enter LP after incubation at 37°C for 2 h (Supplementary Figure S2A). The internalization of PPNs into LP was not much different among the PPNs due to the not much differences of the particle sizes of the PPNs, although pullulan alone entered LP through a diffusion mechanism. To further confirm whether the PPNs were located at the cell surface or were internalized by LP, LP was treated with FITC-PPN3 and the location of FITC-PPN3 was identified by Z-section mode of CLSM. As shown in Figure 3A, the fluorescence intensity of FITC and DAPI was the highest at the center of LP, indicating the internalization of PPNs by LP. We also performed the experiment using a membrane binding dye (FM4-64) as a negative control. As shown in Supplementary Figure S1A, the fluorescence intensity of FITC was the highest at the center of LP, and confirmed that the FITC fluorescence appeared inside the bacteria (Supplementary Figure S1B).

Further studies were performed to determine the effect of incubation temperature and the role of sugar transporters in the internalization of PPNs by LP. To determine if the internalization of PPN3 by LP was temperature-dependent, LP was treated with FITC-PPN3 at 4°C, room temperature or 37°C for 2 h and subsequently analyzed by CLSM and FACS. The internalization of PPN3 by LP was highest at 37°C, suggesting that the internalization of PPN3 was energy-dependent (Supplementary Figure S2B). Furthermore, to determine whether the internalization of PPN3 was via a sugar transporter, LP was pre-treated with 10% (w/v) glucose, galactose, fructose, and PPN3, and then treated with 0.5% (w/v) FITC-PPN3 for 2 h. The internalization was then observed by CLSM and FACS (Figure 3B). The results showed that the internalization of PPN3 was predominantly dependent on the galactose transporter of LP because pre-treatment with galactose blocked approximately 40% of the internalization of PPNs by LP. And it was found that the internalization of PPN3 was also blocked by pre-treatment of PPNs.

### Effects of PPNs on Antimicrobial Activity

**FIGURE 4:**
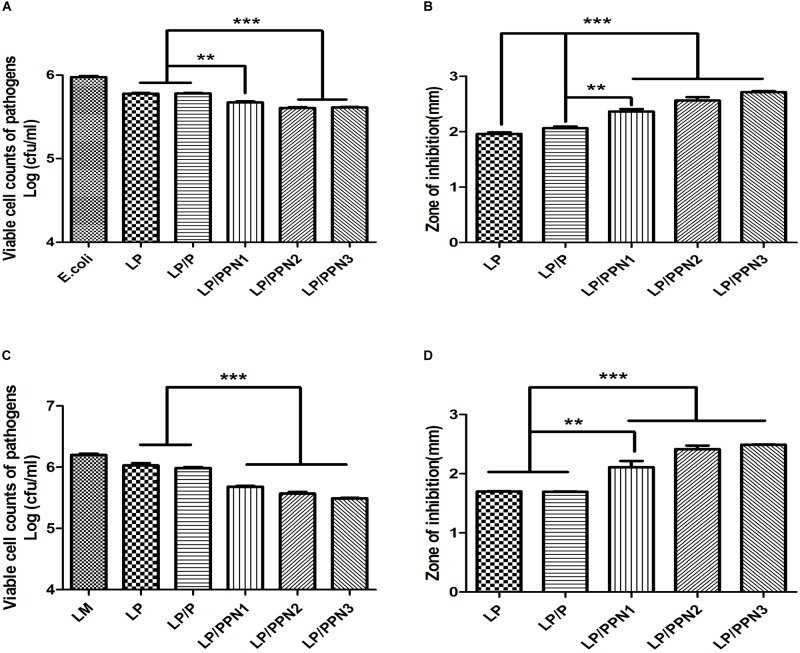
Antimicrobial efficacy of LP/PPNs against *E. coli* and LM **(A-D).** LP treated with PPNs or pullulan were cultured with Gram-negative *E. coli* or Gram-positive LM, and the growth inhibition was calculated by CFU for *E. coli* **(A)** and LM **(C)**. Similarly, the diameters of the growth inhibition of *E. coli* **(B)** and LM **(D)** on LB and BHI agar plates, respectively, were measured. Data are presented as the mean ± SEM of three independent experiments. Statistical significance was analyzed between LP treated with P and LP treated with PPNs by one-way ANOVA and Tukey’s *t*-test (^∗∗^*p* < 0.01 and ^∗∗∗^*p* < 0.001). (LP: *Lactobacillus plantarum*, PPN: phthalyl pullulan nanoparticle, P: pullulan, CFU: colony forming unit, LM: *Listeria monocytogenes*, LB: lysogeny broth, BHI: brain heart infusion).

To evaluate whether the internalization of PPNs by LP affected its antimicrobial activity, LP was treated with three types of PPNs or pullulan itself. The antimicrobial activity of PPN-treated LP (LP/PPNs) was then tested against *E. coli* and LM and compared with that of untreated or pullulan-treated (LP/P) LP. The antimicrobial activity of the LP/PPNs groups was higher than that of untreated LP or LP/P against both *E. coli* and LM by co-culture assays (Figure 4A,C). Interestingly, stronger antimicrobial activity was observed when LP was treated with smaller nanoparticles. To determine whether the PPNs alone had antimicrobial activity, *E. coli* and LM were treated with PPNs. PPNs alone displayed no antimicrobial activity (data not shown), indicating that the antimicrobial activity must be derived from the internalization of the PPNs by LP. In addition, the antimicrobial activity of LP/PPNs against *E. coli* and LM was further evaluated by agar diffusion tests. The inhibition zone was relatively larger when LP was treated with smaller PPNs (Figure 4B,D), suggesting that the agar diffusion tests showed similar antimicrobial patterns of LP/PPNs against *E. coli* and LM.

**FIGURE 5:**
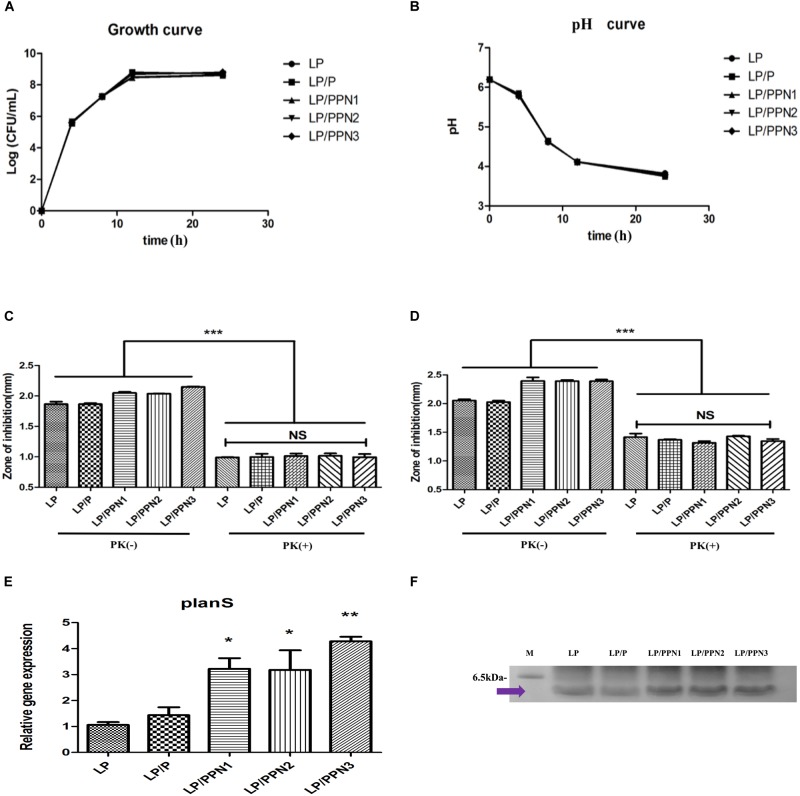
Analysis the mechanism of enhanced antimicrobial ability. Measurement of the growth of LP **(A)** and pH of the culture medium **(B)** among LP groups with internalized PPNs or pullulan. Antimicrobial efficacy of LP/PPNs against *E. coli* **(C)** and LM **(D)** was measured after proteinase K treatment. Relative mRNA expression levels of *planS* compared with 16S rRNA expression levels **(E)**. The isolated plantaricin was determined by SDS–PAGE **(F)**. The full length gel is presented in Supplementary Figure S4. Data are presented as the mean ± SEM of three independent experiments. Statistical significance was analyzed between each group by one-way ANOVA and Tukey’s *t*-test (^∗^*p* < 0.05, ^∗∗^*p* < 0.01, and ^∗∗∗^*p* < 0.001). (LP: *Lactobacillus plantarum*, PPN: phthalyl pullulan nanoparticle, P: pullulan, PK: proteinase K, LM: *Listeria monocytogenes*, SDS–PAGE: sodium dodecyl sulfate polyacrylamide gel electrophoresis).

To test whether LP exerts its antimicrobial activity via plantaricin, a natural peptide, we added proteinase K to the medium of LP during agar diffusion tests. The antimicrobial activity of the proteinase K-treated group was significantly reduced compared with the untreated group (Figure 5C,D), suggesting the degradation of plantaricin by proteinase K.

### Effect of PPNs on Growth and Lactic Acid Production of LP

To test the growth of LP after treatment with PPNs or pullulan, cell colonies were counted at different time points (Figure 5A). The results showed no differences in LP growth with or without PPNs or pullulan treatment. The pH of the culture media of LP after treatment with PPNs or pullulan was also measured to evaluate lactic acid production. Consistent with the growth curve, the pH curve of the LP with or without PPNs or pullulan also showed no significant changes between the groups (Figure 5B). Therefore, the internalization of PPNs by LP had no negative effects on the growth of LP.

### Effect of PPNs on Plantaricin Production by LP

To determine the variations in the production of plantaricin in LP by PPNs, the plantaricin from LP, LP/P, LP/PPNs was isolated and observed by SDS–PAGE. As results, we believed that molecular weight of isolated plantaricin was between from 2.5 to 6.5 kDa because it was already reported by Jimenez-Diaz et al. (1993). Also, the SDS–PAGE showed that LP/PPNs increased the production of plantaricin compared with the LP and LP/P groups under the same isolation conditions (Figure 5F).

### Expression of Stress Response and Plantaricin Genes by Quantitative Real-Time PCR (qRT-PCR)

To evaluate production of plantaricin by PPN-treated LP at the mRNA level, the expression level of plantaricin mRNA in PPN-treated LP was compared with that of untreated LP using qRT-PCR. The plantaricin gene planS was selected, and 16s rRNA was used for normalization. After treatment with PPNs or pullulan for 8 h, the expression level of planS was higher in PPN-treated LP than in untreated or pullulan-treated LP (Figure 5E). The expression level of planS clearly demonstrated the enhanced antimicrobial activity of PPN-treated LP.

**FIGURE 6:**
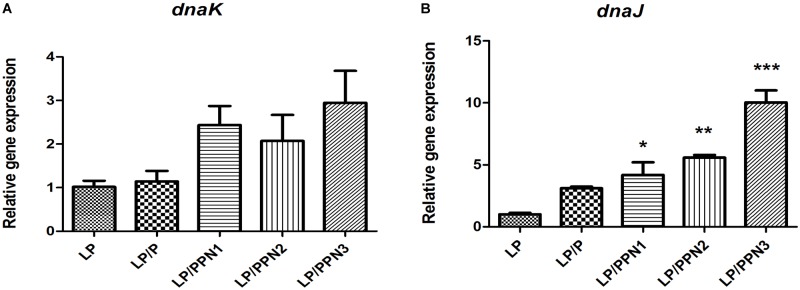
Analysis of genes related to the stress response in LP treated with PPNs. The expression levels of *dnaK* **(A)** and *dnaJ* **(B)** relative to 16s rRNA was quantified by qRT-PCR. Data are presented as the mean ± SEM of three independent experiments. Statistical significance was analyzed between LP and other groups by one-way ANOVA and Tukey’s *t*-test (^∗^*p* < 0.05, ^∗∗^*p* < 0.01, and ^∗∗∗^*p* < 0.001). (LP: *Lactobacillus plantarum*, PPN: phthalyl pullulan nanoparticle, P: pullulan*, dnaK, dnaJ*: heat shock proteins, qRT-PCR: quantitative real-time polymerase chain reaction).

In our previous study, it was found that internalized inulin nanoparticles induced a stress response in PA (Cui et al., 2018; Kim et al., 2018). To verify whether similar behavior occurs in LP, the expression levels of genes related to heat shock proteins, dnaK and dnaJ, were determined. After treatment with PPNs or pullulan for 8 h, the expression levels of dnak and dnaJ were significantly higher in PPN-treated LP than those of untreated LP (Figure 6A,B). The results suggested that the internalization of PPNs by LP induced a stress response.

## Discussion

Many researchers have been interested in the use of probiotics as a promising alternative for synthetic antibiotics because synthetic antibiotics cannot only elicit negative side effects but, with improper use, can also lead to antibiotic-resistant bacteria (Witte, 1998). Prebiotics are generally defined as non-digestible compounds that stimulate the activity and/or growth of probiotics and other microorganisms in the gastrointestinal (GI) tract and have favorable effects on the health of the host (Gibson, 1999), which are often mediated by short chain fatty acids (SCFAs) derived from the metabolism of prebiotics by the gut microbiota (Gourbeyre et al., 2011).

In this study, we developed PPNs as a new formulation of prebiotics to increase the antimicrobial activity of probiotics. The PPNs were prepared by self-assembled nanoparticles after conjugation of hydrophobic phthalic anhydride to hydroxyl groups in pullulan through hydrophobic interactions. It is believed that the reaction occurs between the primary hydroxyl groups of the pullulan and the carboxylic acids of phthalic anhydride through esterification. Furthermore, increased conjugation of phthalic groups resulted in smaller PPN sizes due to the increased hydrophobic interactions among phthalic moieties in the PPNs.

A large number of researchers have been interested in how polymeric nanoparticles are internalized into mammalian cells through endocytosis (Oh and Park, 2014). Because polymeric nanoparticles can deliver therapeutic drugs to the necessary place of action (Zhang et al., 2010) and can be used to overcome cellular barriers when delivering hydrophobic drugs (Blanco et al., 2015); however, research on the internalization of polymeric nanoparticles by prokaryotes as a prebiotic, except for metal nanoparticles, is still in an early stage. Thus far, much of the research on prebiotics has focused on their fermentation by the microbiota (Slavin, 2013). Although the internalization of metal nanoparticles into pathogens occurs via electrostatic interactions (Sanyasi et al., 2016), we sought to develop polymeric nanoparticles as a prebiotic and to elucidate their internalization by probiotics. Interestingly, it is assumed that PPNs enter through galactose transporters on the cell surface of probiotics because pretreatment with galactose significantly decreased the internalization of PPN3 by LP, whereas glucose and fructose inhibited internalization to a lesser degree.

The other goal of our research was to evaluate the effect of prebiotics on the antimicrobial properties of probiotics. Many studies have described the antimicrobial properties of metal nanoparticles against pathogens due to their abrogation of bacterial growth by ionic interactions with the bacterial membrane (Sanyasi et al., 2016). However, metal nanoparticles can cause serious side effects in the host (Roy et al., 2003) and can inhibit both pathogens and beneficial microbes (Travan et al., 2009). Hence, the treatment of LP with PPNs enhanced its antimicrobial activity against both Gram-negative *E. coli* and Gram-positive *L. monocytogenes* compared to pullulan or LP alone although the effect of PPNs internalization in probiotics on observed antimicrobial activities was mild and PPNs did not show toxicity to the host. Particularly, LP/PPN3 showed the highest antimicrobial activity. The results indicated that the increased antimicrobial activity was dependent on the size of the PPNs taken up by LP. Moreover, we made starch and inulin nanoparticles using phthalic acid and the antibacterial activity of these nanoparticles was compared with PPNs (Supplementary Figure S3).

The advantages of probiotics as a food and feed additive have been mostly focused on their antimicrobial properties, suggesting that the enhancement of antimicrobial properties is of importance to probiotics researchers. Plantaricin is a natural peptide produced by LP and was reported to possess strong antimicrobial properties (Nes and Hole, 2000). In this study, treatment of LP with PPNs markedly increased the production of plantaricin, which was confirmed by the mRNA expression of planS. It is hypothesized that the internalization of PPNs directly affected the production of plantaricin. Therefore, we hypothesize that the internalization of PPNs by LP contributes to the enhanced antimicrobial properties of LP via increased expression of the plantaricin.

Notably, probiotics produce bacteriocins as their first defense mechanism (Cleveland et al., 2001; Castro et al., 2015). Several factors, such as culture pH, temperature, and pressure (Kalchayanand et al., 1998; Castro et al., 2015), affect the expression of bacteriocins by the upregulation of genes related to heat shock proteins (HSPs) (Bove et al., 2013) and the stress response. The mRNA expression levels of dnaK and dnaJ in PPN-treated LP were significantly higher than those of untreated LP. The results indicated that the internalization of PPNs by LP induced a mild intracellular stress response to stimulate antimicrobial activities without death of the host. Therefore, the internalization of PPNs by LP enhanced the expression of the plantaricin gene to activate the host’s defense system. Further research is needed to determine the precise mechanism of the internalization of PPNs by LP.

Ultimately, polymeric nanoparticles as prebiotics can exert substantial effects on probiotics, which lead to the increased production of an antimicrobial peptide that is powerful against Gram-positive and Gram-negative pathogens. Therefore, our study shows a new way to produce antimicrobial peptides in probiotics through mild intracellular stimulation by the internalization of PPNs into probiotics, suggesting that PPNs have great promise as an alternative to synthetic antibiotics in veterinary, dairy, and human applications.

## Author Contributions

LH designed and performed the experiments, analyzed the data, and generated all the figures. LH wrote most of the manuscript. W-SK and S-ML discussed the results and corrected the manuscript. S-KK, Y-JC, and C-SC supervised the project.

## Conflict of Interest Statement

The authors declare that the research was conducted in the absence of any commercial or financial relationships that could be construed as a potential conflict of interest.

## Supplementary Material

FIGURE S1 Analysis of the internalization of PPNs by LP. Confocal images and FACS analysis were performed after treatment of LP with 0.5% (w/v) FITC-PPNs for 2 h at 37°C. FITC-PPNs are shown in green, and membrane was stained red with FM4-64. Z-section showed that fluorescence intensity of FITC was highest at the center of LP (A). Confirmed that the FITC fluorescence appeared inside the bacteria (B). (LP: Lactobacillus plantarum, PPN: phthalyl pullulan nanoparticle, CLSM: confocal laser scanning microscopy, FACS: fluorescence-activated cell sorting, FITC: fluorescein isothiocyanate, FM4-64: N-(3-triethylammoniumpropyl)-4-(6-(4-(diethylamino) phenyl) hexatrienyl) pyridinium dibromide).

FIGURE S2 Analysis of the internalization of PPNs by LP. Confocal images and FACS analysis were performed after treatment of LP with 0.5% (w/v) FITC-pullulan and FITC-PPNs for 2 h at 37°C. FITC-PPNs and FITC-pullulan are shown in green, and LP was stained blue with DAPI. The internalization of pullulan and PPNs after 2 h of treatment was quantified by FACS and statistically analyzed **(A)**. Next, LP was treated with 0.5% (w/v) FITC-PPN3 at different temperatures (4, 20, or 37°C) for 2 h **(B)**, and internalization was observed by CLSM and FACS. Confocal and FACS data are representative of three independent experiments, and the average values are presented as the mean ± SEM of three independent FACS experiments in a bar chart. Statistical significance was analyzed between each group by one-way ANOVA and Tukey’s *t*-test (^∗∗∗^*p* < 0.001). Scale bar = 10 μm. (LP: *Lactobacillus plantarum*, PPN: phthalyl pullulan nanoparticle, CLSM: confocal laser scanning microscopy, FACS: fluorescence-activated cell sorting, FITC: fluorescein isothiocyanate, DAPI: 4′,6-diamidino-2-phenylindole).

FIGURE S3 Antimicrobial efficacy of LP, LP/PSNs, LP/PINs, or LP/PPNs against *E. coli K99.* LP treated with different kinds of nanoparticles were cultured with Gram-negative *E. coli.* Statistical significance was analyzed between each group by one-way ANOVA and Tukey’s t-test (^∗∗∗^*p* < 0.001) (LP: *Lactobacillus plantarum*, PPN: phthalyl pullulan nanoparticle, PSN: phthalyl starch nanoparticle, PIN: phthalyl inulin nanoparticle, CFU: colony forming unit).

FIGURE S4 The full length of SDS–PAGE gel. (M: marker, LP: *Lactobacillus plantarum*, PPN: phthalyl pullulan nanoparticle, P: pullulan, SDS–PAGE: sodium dodecyl sulfate polyacrylamide gel electrophoresis).
