# Supplementary figures and images for: Pullulan Nanoparticles as Prebiotics Enhance the Antibacterial Properties of Lactobacillus plantarum Through the Induction of Mild Stress in Probiotics
Source: Front Microbiol. 2019 Feb 6;10:142. doi: 10.3389/fmicb.2019.00142 (PMC6372531; doi:10.3389/fmicb.2019.00142)

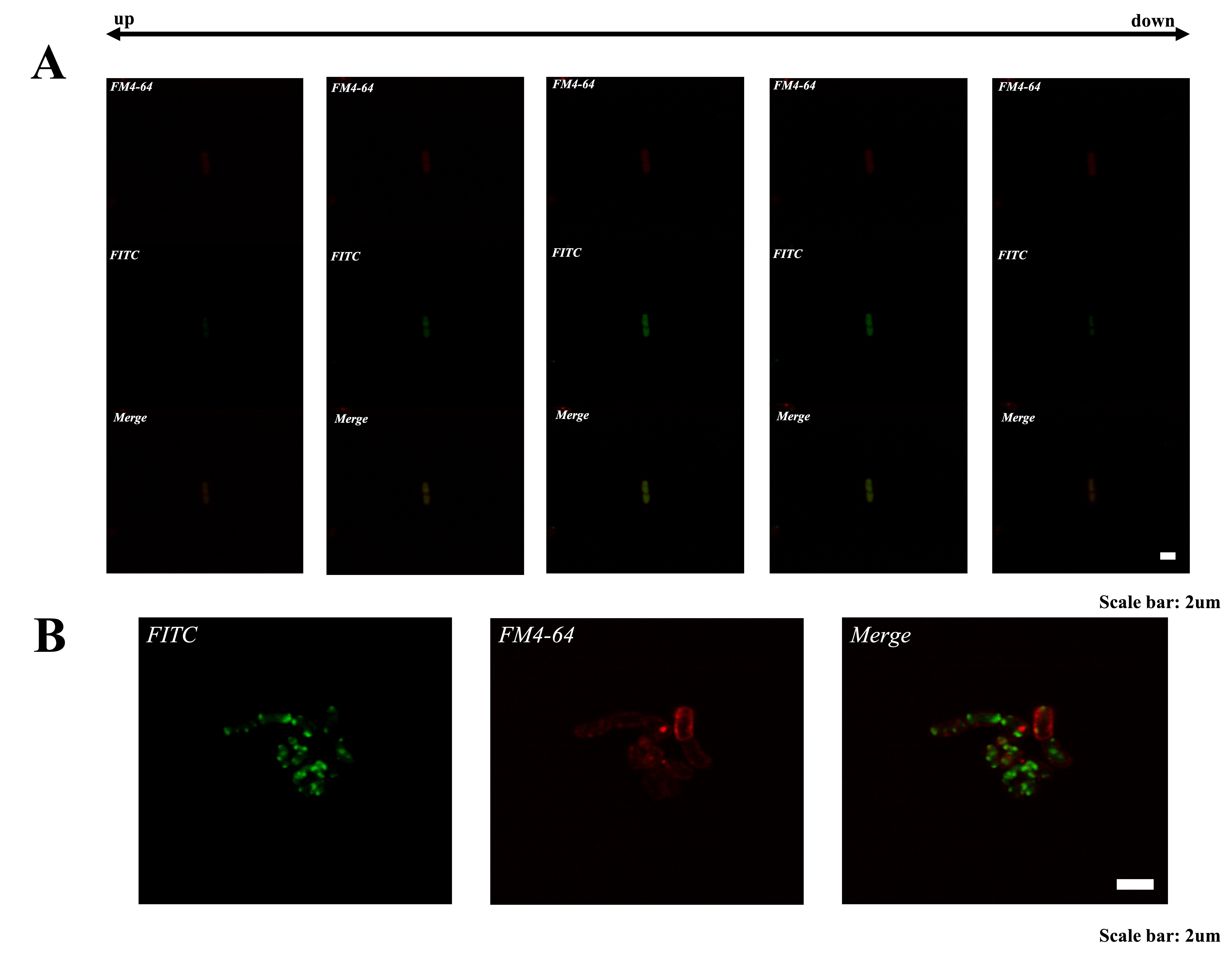

Supplement: FIGURE S1 — Analysis of the internalization of PPNs by LP. Confocal images and FACS analysis were performed after treatment of LP with 0.5% (w/v) FITC-PPNs for 2 h at 37°C. FITC-PPNs are shown in green, and membrane was stained red with FM4-64. Z-section showed that fluorescence intensity of FITC was highest at the center of LP (A). Confirmed that the FITC fluorescence appeared inside the bacteria (B). (LP: Lactobacillus plantarum, PPN: phthalyl pullulan nanoparticle, CLSM: confocal laser scanning microscopy, FACS: fluorescence-activated cell sorting, FITC: fluorescein isothiocyanate, FM4-64: N-(3-triethylammoniumpropyl)-4-(6-(4-(diethylamino) phenyl) hexatrienyl) pyridinium dibromide). [file Image_1.PNG]

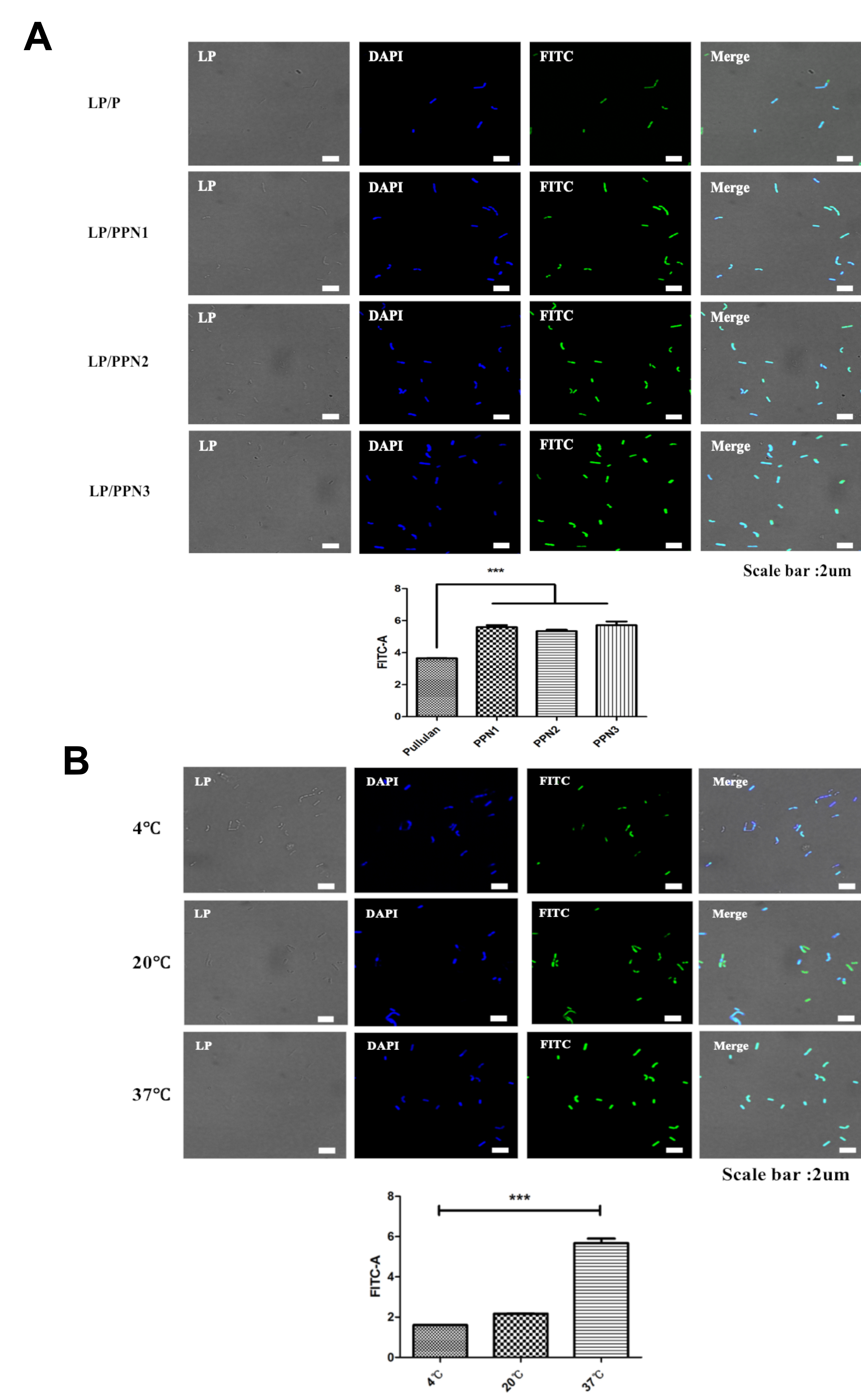

Supplement: FIGURE S2 — Analysis of the internalization of PPNs by LP. Confocal images and FACS analysis were performed after treatment of LP with 0.5% (w/v) FITC-pullulan and FITC-PPNs for 2 h at 37°C. FITC-PPNs and FITC-pullulan are shown in green, and LP was stained blue with DAPI. The internalization of pullulan and PPNs after 2 h of treatment was quantified by FACS and statistically analyzed (A). Next, LP was treated with 0.5% (w/v) FITC-PPN3 at different temperatures (4, 20, or 37°C) for 2 h (B), and internalization was observed by CLSM and FACS. Confocal and FACS data are representative of three independent experiments, and the average values are presented as the mean ± SEM of three independent FACS experiments in a bar chart. Statistical significance was analyzed between each group by one-way ANOVA and Tukey’s t-test (∗∗∗p < 0.001). Scale bar = 10 μm. (LP: Lactobacillus plantarum, PPN: phthalyl pullulan nanoparticle, CLSM: confocal laser scanning microscopy, FACS: fluorescence-activated cell sorting, FITC: fluorescein isothiocyanate, DAPI: 4′,6-diamidino-2-phenylindole). [file Image_2.PNG]

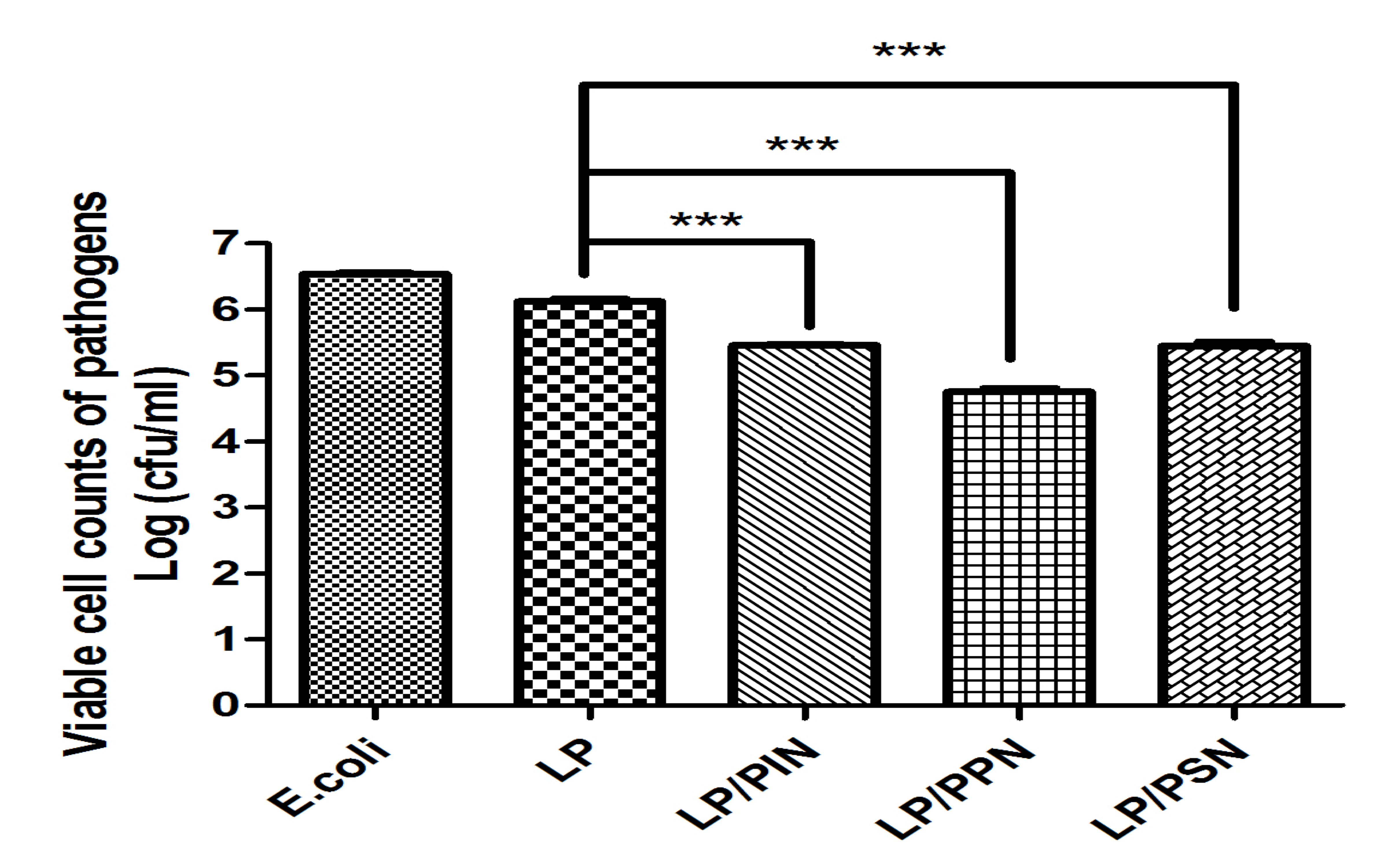

Supplement: FIGURE S3 — Antimicrobial efficacy of LP, LP/PSNs, LP/PINs, or LP/PPNs against E. coli K99. LP treated with different kinds of nanoparticles were cultured with Gram-negative E. coli. Statistical significance was analyzed between each group by one-way ANOVA and Tukey’s t-test (∗∗∗p < 0.001) (LP: Lactobacillus plantarum, PPN: phthalyl pullulan nanoparticle, PSN: phthalyl starch nanoparticle, PIN: phthalyl inulin nanoparticle, CFU: colony forming unit). [file Image_3.PNG]

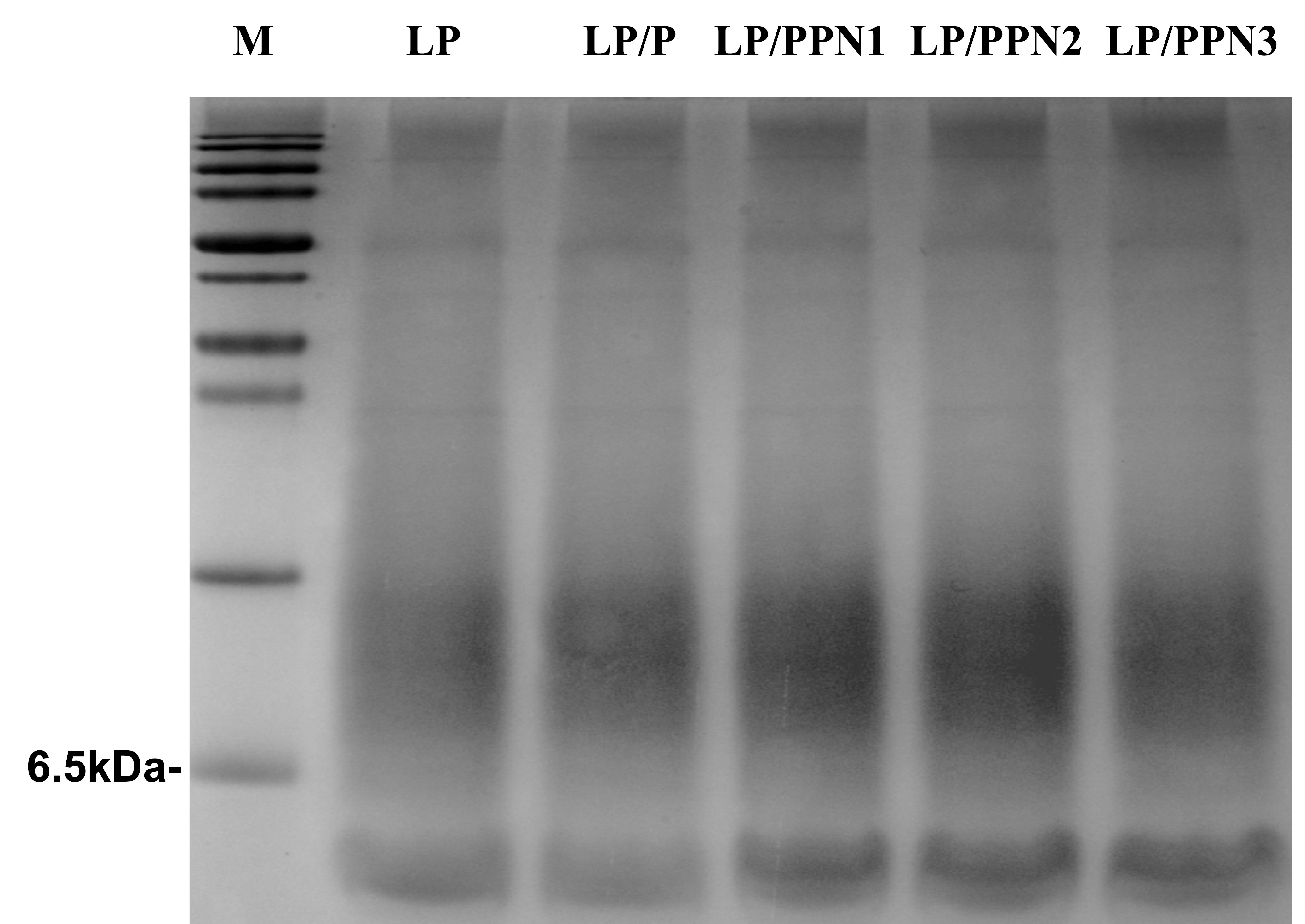

Supplement: FIGURE S4 — The full length of SDS–PAGE gel. (M: marker, LP: Lactobacillus plantarum, PPN: phthalyl pullulan nanoparticle, P: pullulan, SDS–PAGE: sodium dodecyl sulfate polyacrylamide gel electrophoresis). [file Image_4.PNG]
